# Site-level progression of periodontal disease during a follow-up period

**DOI:** 10.1371/journal.pone.0188670

**Published:** 2017-12-04

**Authors:** Yoshiaki Nomura, Toshiya Morozumi, Taneaki Nakagawa, Tsutomu Sugaya, Masamitsu Kawanami, Fumihiko Suzuki, Keiso Takahashi, Yuzo Abe, Soh Sato, Asako Makino-Oi, Atsushi Saito, Satomi Takano, Masato Minabe, Yohei Nakayama, Yorimasa Ogata, Hiroaki Kobayashi, Yuichi Izumi, Naoyuki Sugano, Koichi Ito, Satoshi Sekino, Yukihiro Numabe, Chie Fukaya, Nobuo Yoshinari, Mitsuo Fukuda, Toshihide Noguchi, Tomoo Kono, Makoto Umeda, Osamu Fujise, Fusanori Nishimura, Atsutoshi Yoshimura, Yoshitaka Hara, Toshiaki Nakamura, Kazuyuki Noguchi, Erika Kakuta, Nobuhiro Hanada, Shogo Takashiba, Yasuharu Amitani, Hiromasa Yoshie

**Affiliations:** 1 Department of Translational Research, Tsurumi University School of Dental Medicine, Yokohama, Japan; 2 Division of Periodontology, Department of Oral Biological Science, Niigata University Graduate School of Medical and Dental Sciences, Niigata, Japan; 3 Department of Dentistry and Oral Surgery, School of Medicine, Keio University, Tokyo, Japan; 4 Division of Periodontology and Endodontology, Department of Oral Health Science, Hokkaido University Graduate School of Dental Medicine, Sapporo, Japan; 5 Division of Dental Anesthesiology, Department of Oral Surgery, Ohu University School of Dentistry, Kooriyama, Japan; 6 Division of Periodontics, Department of Conservative Dentistry, Ohu University School of Dentistry, Kooriyama, Japan; 7 Comprehensive Dental Care, The Nippon Dental University Niigata Hospital, Niigata, Japan; 8 Department of Periodontology, School of life Dentistry at Niigata, The Nippon Dental University, Niigata, Japan; 9 Department of Periodontology, Tokyo Dental College, Tokyo, Japan; 10 Bunkyo-Dori Dental Clinic, Chiba, Japan; 11 Division of Periodontology, Department of Oral Interdisciplinary Medicine, School of Dentistry, Kanagawa Dental University, Yokosuka, Japan; 12 Department of Periodontology, Nihon University School of Dentistry at Matsudo, Matsudo, Japan; 13 Department of Periodontology, Graduate School of Medical and Dental Sciences, Tokyo Medical and Dental University, Tokyo, Japan; 14 Department of Periodontology, Nihon University School of Dentistry, Tokyo, Japan; 15 Nihon University School of Dentistry, Tokyo, Japan; 16 Department of Periodontology, School of Life Dentistry at Tokyo, The Nippon Dental University, Tokyo, Japan; 17 Department of Periodontology, School of Dentistry, Matsumoto Dental University, Shiojiri, Japan; 18 Department of Periodontology, School of Dentistry, Aichi Gakuin University, Nagoya, Japan; 19 Department of Periodontology, Osaka Dental University, Osaka, Japan; 20 Section of Periodontology, Division of Oral Rehabilitation, Faculty of Dental Science, Kyushu University, Fukuoka, Japan; 21 Department of Periodontology, Unit of Translational Medicine, Nagasaki University Graduate School of Biomedical Sciences, Nagasaki, Japan; 22 Department of Periodontology, Kagoshima University Graduate School of Medical and Dental Sciences, Kagoshima, Japan; 23 Department of Oral Microbiology, Tsurumi University School of Dental Medicine, Yokohama, Japan; 24 Department of Pathophysiology-Periodontal Science, Okayama University Graduate School of Medicine, Dentistry and Pharmaceutical Sciences, Okayama, Japan; 25 Department of Mathematics, Tsurumi University School of Dental Medicine, Yokohama, Japan; University of Washington, UNITED STATES

## Abstract

Periodontal disease is assessed and its progression is determined via observations on a site-by-site basis. Periodontal data are complex and structured in multiple levels; thus, applying a summary statistical approach (i.e., the mean) for site-level evaluations results in loss of information. Previous studies have shown the availability of mixed effects modeling. However, clinically beneficial information on the progression of periodontal disease during the follow-up period is not available.

We conducted a multicenter prospective cohort study. Using mixed effects modeling, we analyzed 18,834 sites distributed on 3,139 teeth in 124 patients, and data were collected 5 times over a 24-month follow-up period. The change in the clinical attachment level (CAL) was used as the outcome variable. The CAL at baseline was an important determinant of the CAL changes, which varied widely according to the tooth surface. The salivary levels of periodontal pathogens, such as *Porphyromonas gingivalis* and *Aggregatibacter actinomycetemcomitans*, were affected by CAL progression. “Linear”- and “burst”-type patterns of CAL progression occurred simultaneously within the same patient. More than half of the teeth that presented burst-type progression sites also presented linear-type progression sites, and most of the progressions were of the linear type. Maxillary premolars and anterior teeth tended to show burst-type progression. The parameters identified in this study may guide practitioners in determining the type and extent of treatment needed at the site and patient levels. In addition, these results show that prior hypotheses concerning "burst" and "linear" theories are not valid.

## Introduction

Periodontal disease is a widespread chronic inflammatory disease caused by the host immunoresponse and periodontal pathogens. Periodontal therapeutic treatments consist of active therapy and subsequent follow-up examinations. Longitudinal studies have shown that periodontal conditions progress during follow-up. A slight increase in the pocket probing depth (PPD) [1–6], loss of the clinical attachment level (CAL) [2, 3] and loss of teeth [4, 7, 8] have been reported. Previous studies have also provided useful information on the risk factors for the progression of this disease. At the subject level, smoking, compliance with treatment, diabetes mellitus, age [9], and proportion of deep PPD [10, 11] were associated with CAL loss. Moreover, sites with PPD≥6 mm were at a significantly higher risk of CAL loss [10].

One of the important features of periodontal diseases is the site-specific localization of infectious processes that lead to tissue destruction [12]. Assessments of disease progression are performed via observations and measurements on a site-by-site basis. The progression may vary for different sites on a tooth and be affected by properties of the tooth, and it may also be concomitant to the risk factors at the subject level. Therefore, in follow-up examinations, the clinical interest is in the behavior at each site.

Periodontal data are complex and structured in multiple levels (usually three or four), such as the individual-level, tooth-level, site-level and repeated-level measurements. Summary statistical approaches (i.e., mean or sum scores) or the maximum value of the site-level evaluation have been applied for patient-level evaluations. However, aggregating site-level information causes a loss of information [13].

When several levels are involved in one statistical model, site-level observations are not independent because they are nested within the tooth level and patient level. Traditional statistical models tend to overlook multilevel structures and disturbed independency among observations, which leads to type I errors [14] and potential misinterpretations. Evaluations of periodontal disease progression should consider the level hierarchy using analytical models; thus, mixed effects models have been introduced and employed to properly assess multilevel-structured data.

By applying a mixed effects model for periodontal data, valuable information has been obtained on periodontal disease progression. Age and plaque scores affected the bone height [15, 16]. Dental plaque is considered at the site level, tooth mobility is considered at the tooth level and several factors, such as age and socio-economic status, are considered at the patient level, and they affected changes in the PPD [17]. Tooth position affected the loss of CAL [18, 19]. The "linear" and "burst" theories of periodontal disease progression represent manifestations of the same phenomenon [19]. The prognostic factors at the bone level at baseline were tooth mobility, bone level at a site, and PPD at a site [20]. However, the outcome variables varied, and information regarding CAL loss was limited. Tooth loss is a true endpoint for long-term evaluations; however, in short-term observations, CAL loss is a surrogate major endpoint for follow-up therapy.

As described above, previous studies have applied mixed effects models and generated valuable information for clinical practice. However, important information about periodontal disease remains unknown, such as the sites that are predisposed to progression, the risk factors that affect progression and the estimated values of disease progression. Therefore, the aims of this study were to identify the characteristics of sites with CAL loss over a 24-month follow-up period after active periodontal therapy, and the evaluations included the linear and burst theories of periodontal disease progression.

## Methods

### Study design

#### Setting

This study was performed as a clinical research project for the diagnosis of periodontitis by the Japanese Society of Periodontology. In cooperation with 17 facilities (16 university hospitals and one clinic) in Japan, 163 follow-up patients with chronic periodontitis who had finished active treatment regulated by the Japanese Insurance system were registered between February 2009 and February 2012.

Briefly, regulated periodontal treatment consists of 1) Periodontal examination at the first visit, 2) Full-mouth supragingival scaling, 3) Periodontal examination as reevaluation, 4) Subgingival scaling and root planing at sites with a probing depth > 4 mm or a probing depth = 4 mm with BOP, 5) Periodontal examination as reevaluation and periodontal surgery if necessary, and 6) Follow-up. In this study, no patients underwent periodontal surgery.

#### Diagnosis

Each diagnosis was based on the Guidelines of the American Academy of Periodontology [21]. Oral examinations were carried out by one examiner at each institute (T.M., C.F., T.S., K.T., Y.A., A.M., M.M., Y.N., H.K., N.S., S.S., N.Y., M.F., T.K., O.F., A.Y., and T.N.). All examiners were periodontists licensed by the Japanese Society of Periodontology.

#### Patient

All patients were ≥ 30 years of age, systemically healthy, possessed at least 20 teeth and had not taken systemic antibiotics, anti-inflammatory drugs or immunosuppressive drugs in the 3 months before the start of the investigation. Before periodontal treatment and the initiation of this study, the systemic health conditions of the patients were assessed by questionnaires and medical interviews. All patients were systemically healthy.

#### Sample size

The optimal sample size required to detect statistically significant differences in *P*. *gingivalis* ratios (%) between patients with or without progression during the supportive periodontal therapy period was determined; based on our previous study [22], we calculated that 77 patients were necessary for each group (α = 0.05 and β = 0.80).

#### Drop out

Of the initial 163 follow-up patients, 39 (23.9%) failed to complete the program during the study period. Fourteen patients dropped out following the Great East Japan Earthquake (three facilities), nine failed to comply with visits (six facilities) and eight dropped out for personal reasons (four facilities). Others withdrew because of the use of antimicrobial agents for acute periodontal abscesses (seven patients in five facilities) and because of tooth extraction for root fracture (one patient). In total, 124 subjects successfully completed the study protocol. These 124 patients level analysis are described in our previous report [23, 24].

### Research data

The following variables were recorded at baseline and after 6, 12, 18 and 24 months. All data are presented in S1 Table. Descriptive analyses of the variables are shown in S2 Table.

#### Subject-level explanatory variables

The demographic background of the patients, such as the age, gender and smoking status, were obtained at baseline. The salivary levels of the total bacteria, *Porphyromonas gingivalis* and *Aggregatibacter actinomycetemcomitans* were measured using the Invader PLUS Assay [25, 26]. As shown in S1 Table, bacterial levels showed a skewed distribution. Bacterial variables were dichotomized based on previous reports [22, 27–29]. To differentiate sites based on whether they had more or less than 3 mm of CAL progression, cut-off values were set to minimize the difference between sensitivity and specificity [22–24, 29]. The cut-off value of *A*. *actinomycetemcomitans* at baseline was 0.00006% of Total bacteria (Sensitivity: 0.24, Specificity: 0.86), while that of *P*. *gingivalis* was 0.0067% (Sensitivity: 0.58, Specificity: 0.67).

Only one patient was a current smoker (20 cigarettes/day, 25 yrs), and three were previous smokers (5 cigarettes/day, 30 yrs; 10 cigarettes/day, 20 yrs; 10 cigarettes/day, 30 yrs); thus, smoking status was not used as an independent variable.

#### Tooth-level explanatory variable

Tooth mobility was evaluated using the standard procedure as described in periodontology textbooks [30, 31]. The assessments were classified as follows:

Degree 1: Mobility of the crown of the tooth, 0.2–1 mm in the horizontal direction
Degree 2: Mobility of the crown of the tooth exceeding 1 mm in horizontal direction
Degree 3: Mobility of the crown of tooth in the vertical direction

Because tooth mobility level 3 was only present in one tooth, levels 2 and 3 were combined.

The plaque index (PlI) was recorded at four sites per tooth: mesial, buccal, distal and lingual.

The mean for each tooth was used for the tooth-level variable [31].

#### Site-level explanatory variable

The CAL and bleeding on probing (BOP) were recorded at six sites per tooth (mesiobuccal, buccal, distobuccal, mesiolingual, lingual and distolingual). The tooth surfaces were reclassified into eight categories, which included combinations of maxillary or mandibular and buccal (labial), palatal (lingual), approximal or distal. In this study, third molars were included to distinguish whether the distal surface of the 2^nd^ molar is approximal or distal.

The final data consisted of 18,834 sites distributed on 3,139 teeth in 124 patients, and these data were collected 5 times throughout the study period.

#### Outcome variables

The CAL (mm) and differences in the CAL between the baseline and after 24 months were used as outcome variables (ΔCAL). Because a change in CAL ≥ 3 mm is a generally accepted threshold for the progression of CAL [27, 28, 32, 33], he changes over 24 months were classified into six categories: a difference of ≤ -3 mm was defined as improved, between -3 mm and -2 mm was defined as slightly improved, between -1 mm and 1 mm was defined as stable, between 1 mm and 2 mm was defined as slightly progressed, and ≥ 3 mm was defined as progressed. In addition, sites with both ≤ -3 mm improvement and ≥ 3 mm progression were defined as fluctuated.

### Multilevel modeling

Periodontal data in this study consisted of a three-level hierarchical structure: patient level, tooth level and site level. The salivary levels of periodontal pathogens, such as *A*. *actinomycetemcomitans* and *P*. *gingivalis*, were treated as patient-level data. Tooth mobility and PlI were treated as tooth-level data. Clinical parameters with or without BOP, CAL at baseline, tooth type, type of tooth surface and the combination of tooth type and surface were treated as site-level data. P-values less than 0.05 were considered statistically significant.

According to the properties of the outcome variables and analyses, file layouts were changed for each analysis. Three data sets were used for the mixed effects modeling. For the first data set (Model 1), the difference in CAL between the baseline and after 24 months (ΔCAL) was used as the outcome variable, baseline data were used as the explanatory variables, and patient and tooth data were used for the grouping variables.

For the second data set (Model 2), the patterns of CAL changes were analyzed using the 5 CAL measurements as the outcome variables, and the 5 measurements of clinical parameters and salivary levels of periodontal pathogens as explanatory variables. A mixed effects model was constructed using the repeated measures design. For CAL values, a Gamma distribution was chosen as the most suitable option for probability distribution. For this analysis, time was an independent variable. Therefore, all variables were reconstituted by the site and time. Therefore, the total number of samples was 94,170 (n = 41690, 28005 and 24475 for CAL at baseline < 3 mm, 3 mm and > 3 mm, respectively).

For the final data set (Model 3), variables that affect the progression type were analyzed. Before performing mixed effects modeling, a hierarchical cluster analysis of the variables was performed using all 5 measured CAL values. The generated clusters were reclassified into burst- or linear-type progression. Dichotomous variables of these two burst or linear types were used as the outcome variables. A multilevel logistic regression analysis was performed by applying generalized mixed effects modeling. For this analysis, sites that showed burst- or linear type progression were selected from the previous data set, resulting in a sample size of 3440 (Burst type = 564, Linear type = 2875).

All analyses were performed using SPSS Statistics version 22.0 (IBM, Tokyo, Japan).

Models used in this study are described in S1 File.

### Ethical approval

The study was conducted in compliance with the principles outlined in the Helsinki Declaration. Informed written consent was obtained from each subject, and the protocol was approved by the Institutional Review Board of each participating institution. The names of the ethics committee members and their reference numbers are listed in S3 Table.

## Results

Among the 18,834 sites investigated in this study, 202 sites (1.1%) were improved, 18,272 sites (97.0%) were stable, and 360 (1.9%) sites were progressed during the 24-month follow-up period. At the patient level, 40 (32.3%) patients had only stable sites, 21 (16.9%) had stable and improved sites, 30 (24.2%) had stable and progressed sites, and 33 (26.7%) had stable, improved and progressed sites. At the tooth level, most of the teeth (89.2%) had only stable sites, and only 0.4% of the teeth had both improved and progressed sites. At the site level, the proportions of progressed sites were higher in the maxillary molars than in the other teeth. The descriptive statistics of the differences in CAL between the baseline and after 24 months (ΔCAL) are detailed in S4 Table.

By applying mixed effects modeling, we first analyzed ΔCAL as a dependent variable. When PlI and BOP were included in the fixed effects model, they were not statistically significant and were thus removed from the random effects model. The fixed effects model with all variables at baseline is shown in S5 Table. Table 1 demonstrates the explanatory variables that were significantly associated with ΔCAL in a random intercept model.

**Table 1:** Multilevel random intercept model for changes in CAL between the baseline and after 24 months (Model 1).

			Coefficient	95% CI		P-value
				Lower	Upper	
Intercept			0.965	0.856	1.074	<0.001
**Subject-level explanatory variable**						
Salivary levels of *A*. *a*		<0.00006%	Reference			
		0.00006%<	0.264	0.008	0.519	0.043
Salivary levels of *P*. *g*		<0.0067%	Reference			
		0.0067%<	0.174	0.026	0.321	0.021
**Tooth-level explanatory variable**						
Tooth mobility		0	Reference			
		1	0.367	0.285	0.449	<0.001
		2–3	0.840	0.592	1.088	<0.001
**Site-level explanatory variable**						
CAL at Baseline			-0.436	-0.448	-0.423	<0.001
Mandibular	Anterior	Lingual	Reference			
		Labial	0.122	0.042	0.201	0.003
		Approximal	0.171	0.108	0.234	<0.001
	Premolar	Lingual	0.098	-0.009	0.204	0.073
		Buccal	0.214	0.107	0.321	<0.001
		Approximal	0.251	0.163	0.338	<0.001
	Molar	Lingual	0.428	0.317	0.539	<0.001
		Buccal	0.354	0.243	0.465	<0.001
		Approximal	0.433	0.340	0.527	<0.001
		Distal	0.450	0.342	0.558	<0.001
Maxillary	Anterior	Paratal	-0.117	-0.211	-0.022	0.016
		Labial	-0.020	-0.115	0.075	0.677
		Approximal	0.146	0.066	0.227	0.000
	Premolar	Palatal	0.116	0.009	0.223	0.034
		Buccal	0.241	0.134	0.348	<0.001
		Approximal	0.365	0.277	0.453	<0.001
	Molar	Palatal	0.634	0.520	0.747	<0.001
		Buccal	0.760	0.646	0.873	<0.001
		Approximal	0.701	0.605	0.797	<0.001
		Distal	0.642	0.533	0.751	<0.001

CAL: clinical attachment level; *A*. *a*: *Aggregatibacter actinomycetemcomitans*; *P*. *g*: *Porphyromonas gingivalis*

Both periodontal pathogens were statistically significant. The coefficients of CAL at the baseline were all negative. Almost all coefficients of the tooth surfaces were statistically significant when the lingual surface of the mandibular anterior tooth was used as a reference. The coefficient of the buccal surface of the maxillary molar teeth was the highest. The value of ΔCAL appeared to vary between the tooth surfaces, and the CAL value at baseline distinctively affected the ΔCAL value. The mean predictive values were largely consistent with the observed values (S6 Table).

For the variables of CAL at baseline and tooth type, random slopes were included in the models. Results are shown in S7 Table. The random slope of CAL at baseline was statistically significant. All tooth types were statistically significant when the mandibular anterior tooth was used as a reference. These results indicated that ΔCAL varies according to the CAL at baseline, and the random slope for molars was different from those of premolars and anterior teeth.

**Fig 1:**
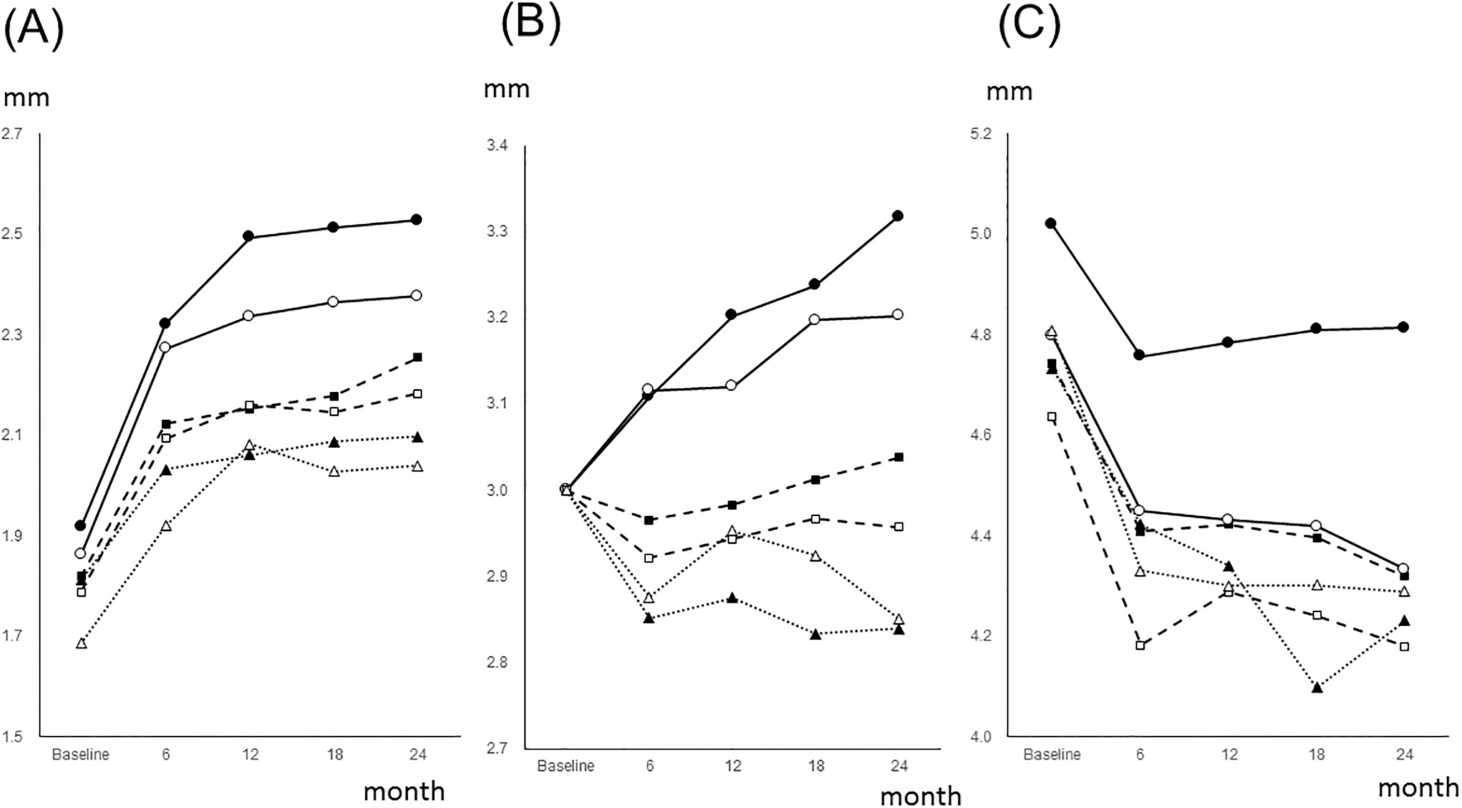
Mean values of the CAL changes during the 24-month follow-up period. CAL changes during the 24-month follow-up period are separately illustrated by the CAL at baseline and by the type of tooth surface. Baseline CAL values are divided into three groups: (A) <3mm; (B) 3 mm; and (C) > 3 mm. ─●─: Maxillary molar, ---■---: Maxillary premolar, ···▲···: Maxillary anterior, ─○─: Maxillary molar, ---□---: Maxillary premolar, ···△···: Maxillary anterior Baseline CAL values of < 3mm gradually deteriorated, while baseline CAL values of > 3 mm improved. Molars with a baseline CAL of 3 mm progressed, whereas premolars and anterior teeth were stable or improved. CAL: clinical attachment level.

The CAL changes are illustrated separately by combinations of tooth types and the CAL at baseline (Fig 1). A baseline CAL of < 3 mm showed gradual progression, while a baseline CAL of > 3 mm showed improvement. Molars with a baseline CAL of 3 mm were progressed, whereas premolars and anterior teeth were stable or improved. Therefore, because the CAL behavior depended on the CAL at the baseline, mixed effects models were constructed individually using the baseline CAL.

The CAL changes were analyzed by the mixed effects model with repeated measures. The tooth surface was used as explanatory variables. For the groups of CAL at baseline < 3 mm and > 3 mm, random intercept was included for the tooth level. For the group of CAL at baseline = 3 mm, random slope was included for the tooth type. The results are listed in Table 2.

**Table 2:** Multilevel random effects model with repeated measures for (Model 2 (A), (B) and (C)).

		Model 2 (A), CAL<3mm (Random Intercept)				Model 2 (B), CAL = 3mm Random Slope				Model 2 (C), CAL>3mm Random Intercept			
		Coefficient	95% CI		P-value	Coefficient	95% CI		P-value	Coefficient	95% CI		P-value
			Lower	Upper			Lower	Upper			Lower	Upper	
Intercept		0.512	0.495	0.529	<0.001	1.067	1.053	1.082	<0.001	1.563	1.538	1.588	<0.001
Time		0.046	0.044	0.049	<0.001	0.002	0	0.004	0.124	-0.023	-0.025	-0.02	<0.001
Tooth Type													
Mandibular	Anterior	Reference				Reference				Reference			
	Premolar	0.060	0.038	0.082	<0.001	0.013	-0.006	0.032	0.174	-0.011	-0.043	0.022	0.508
	Molar	0.126	0.102	0.150	<0.001	0.065	0.047	0.084	<0.001	0.009	-0.022	0.039	0.580
Maxillary	Anterior	0.042	0.022	0.061	<0.001	-0.011	-0.027	0.006	0.204	-0.020	-0.053	0.012	0.225
	Premolar	0.083	0.060	0.106	<0.001	0.028	0.011	0.046	0.001	-0.001	-0.034	0.032	0.932
	Molar	0.186	0.160	0.212	<0.001	0.081	0.063	0.099	<0.001	0.096	0.064	0.127	<0.001

CAL: clinical attachment level

For groups with a baseline CAL > 3 mm, the coefficient of time was negative, which indicated that sites with CAL > 3 mm at baseline were generally improved. For the group with baseline CAL of 3 mm (Model 2 (B)), the coefficients of mandibular molar, maxillary premolar and maxillary molar were statistically significant. This indicated that for these teeth, the slope of their CAL changes across 24 months were different from that of mandibular anterior teeth. In addition, mandibular and maxillary molars had the highest coefficients of all teeth. These results are consistent with Fig 1. The fixed effects models for all explanatory variables used in this study are shown in S8 Table. When tooth type was combined with tooth surface and analyzed similarly to Model 1 (Table 1), the maxillary molar coefficient was higher than those of other tooth surfaces in the random effects models. These results are shown in S9 Table.

**Fig 2:**
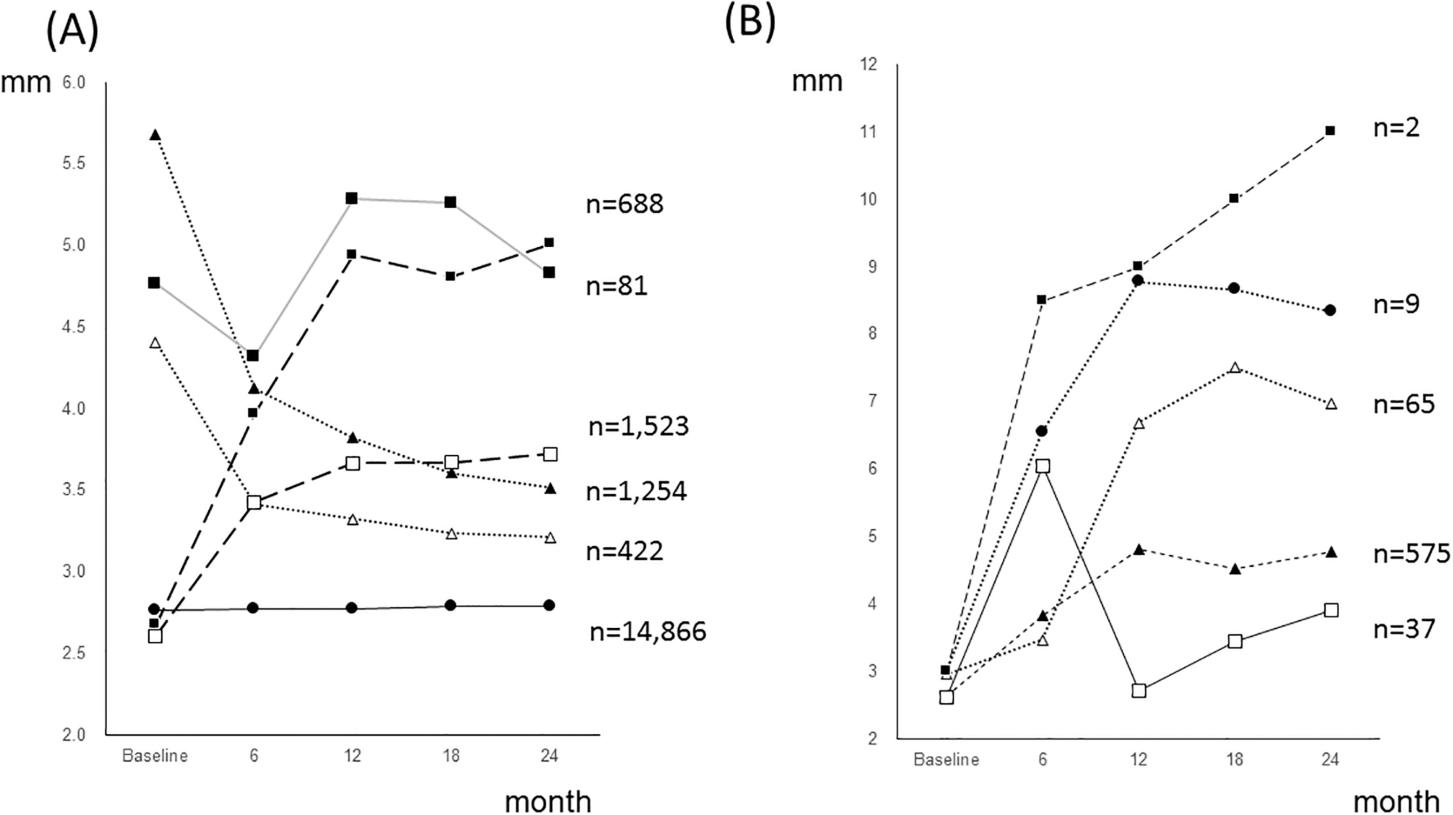
CAL change patterns during the 24-month follow-up period. (A) Changes of the improved, slightly improved, stable, slightly progressed, progressed and fluctuated categories.···▲···: Improved, ··△···: Slightly improved, ─●─: Stable.---□---: Slightly progressed, ---■---: progressed, ─■─: FluctuatedDifferences in the CAL changes over 24 months were classified into six categories: ≤ -3 mm, improved; between -3 mm and -2 mm, slightly improved; between -1 mm to 1 mm, stable; between 1 mm and 2 mm, slightly progressed; 3mm, progressed. In addition, cases with both ≤ -3 mm and ≥ 3mm were classified as fluctuated.(B) CAL progression patterns of the progressed category···▲···: Cluster 1, ···△···: Cluster 2, ─●─: Cluster 3.─□─: Cluster 4, —■—: Cluster 5A hierarchical cluster analysis was performed for the progressed type portrayed in Fig. 2(A), and 5 clusters were generated. The slope of cluster 1 was moderate, and the slopes of the other clusters were steep. Cluster 1 may correspond to the linear-type progressed sites, and the other clusters may correspond to the burst-type progressed sites.

Finally, we classified the CAL changes during the 24-month follow-up period into six groups: improved, slightly improved, stable, slightly progressed, progressed and fluctuated, which accounted for 422 (2.2%), 1254 (6.7%), 14866 (78.9%), 1523 (8.1%) 688 (3.7%) and 81 (0.4%) changes, respectively. For the progressed group, a cluster analysis was performed that generated 5 clusters, which accounted for 575 (86.3%), 65 (9.4%), 9 (1.3%), 37 (5.4%) and 2 (0.3%) changes. The changes of these six groups and 5 clusters are portrayed in Fig 2(A) and 2(B). The slope of cluster 1 was moderate, and the slopes of the other clusters were steep. Cluster 1 may correspond to linear-type progressed sites, and the other clusters may correspond to burst-type progressed sites. Cross-tabulations by the number of linear- and burst-type progressed sites at the patient level and the tooth level are shown in S10 Table. Among the 83 patients with at least one progressed site, 43 (51.8%) had only a linear-type progressed site and 40 (51.8%) had both linear- and burst-type progressed sites. One patient had a burst-type progressed site without a linear type. Among the 71 teeth with a burst-type progression site, 32 (45.1%) lacked a linear progression site and 39 (54.9%) had an accompanying linear-type progression site. To investigate the predictors for distinguishing between linear-type and burst-type progression, a multilevel logistic regression analysis with repeated measures was performed. The fixed effects model to distinguish burst-type from linear-type progression using all the independent variables used in this study is shown in S11 Table. Table 3 shows the results of the random intercept model. The buccal surface of maxillary premolars and maxillary anterior teeth were statistically significant variables, and their coefficients were positive. The coefficients of tooth mobility were statistically significant, and their coefficients were negative.

**Table 3:** Multilevel logistic regression model with repeated measures to distinguish “linear” and “burst” progression during the 24-month follow-up period.

			Coefficient	95% CI		P-value
				Lower	Upper	
Intercept			5.233	3.824	6.643	<0.001
Time			<0.001	0.000	0.000	>0.999
Tooth mobility		0	Reference			
		1	-1.123	-1.728	-0.518	<0.001
		2–3	-1.914	-2.688	-1.140	<0.001
Mandibular	Anterior	Lingual	Reference			
		Labial	0.172	-0.597	0.940	0.661
		Approximal	-0.051	-0.684	0.581	0.873
	Premolar	Lingual	-0.354	-2.523	1.814	0.749
		Buccal	0.018	-2.072	2.109	0.986
		Approximal	-0.515	-2.531	1.501	0.617
	Molar	Lingual	-1.676	-3.459	0.108	0.066
		Buccal	-0.888	-2.681	0.904	0.331
		Approximal	-1.765	-3.527	-0.002	0.050
		Distal	-1.609	-3.385	0.168	0.076
Maxillary	Anterior	Lingual	-1.478	-3.572	0.616	0.167
		Labial	3.414	0.608	6.221	0.017
		Approximal	-0.868	-2.833	1.098	0.387
	Premolar	Palatal	6.276	3.056	9.497	<0.001
		Buccal	1.109	-1.201	3.419	0.347
		Approximal	-0.724	-2.644	1.195	0.460
	Molar	Palatal	0.578	-1.226	2.382	0.530
		Buccal	-0.304	-2.100	1.493	0.740
		Approximal	-0.532	-2.294	1.229	0.553
		Distal	-1.001	-2.780	0.778	0.270

CAL: clinical attachment level

## Discussion

The characteristics of periodontal disease represent heterogeneous phenomena, and the clinical manifestations of the disease caused by interactions between the host and agents may vary according to the site and tooth and may even vary within the same individual and longitudinally over time [33, 34]. Studies of the associations between clinical or etiological parameters and the progression of periodontal disease during follow-up have been conducted at the patient level, tooth level and site level. At the patient level, aggregated summary measurements of site-level information have been utilized and were found to result in a loss of information and subsequent misinterpretation. Furthermore, standard procedures for examining periodontal tissue rely on a site-based examination, and the management of a periodontal lesion is predominantly site specific [35]. Another interest of dental clinicians is the level of disease and its behavior at each site because of the site-specific nature of periodontal disease [36–40]. Most sites are inactive and do not show progression. In fact, the number of stable sites in this study was 18,272 (97.0%).

As shown in S3 Table, even using a primitive descriptive analysis and cross-tabulation, a site-level analysis provides beneficial information. At the patient level, 32.3% of the patients had only stable sites, and 26.7% of the patients had both improved and progressed sites during the 24-month follow-up period. At the tooth level, most of the teeth were stable, and an extreme minority (0.4%) had both improved and progressed sites. Progressed and improved teeth were observed in only 6.4% and 3.4% of the patients, respectively. These data indicate that in approximately one-quarter of the patients, progression and improvement proceed simultaneously in the same individual. Therefore, the classification of patients as progressive, stable, or improved may be elusive. At the tooth level, most teeth were stable, and progressed teeth were a small minority. However, in dental practice, identifying these minority downhill teeth or sites is crucial for predicting the disease progression using clinical parameters and efficiently managing the etiological agents.

Multilevel modeling is one of the techniques of mixed effects analyses and has been employed for periodontal data. Previous studies have specified the risk factors that are associated with the progression of periodontal conditions at the patient, tooth and site levels. Novel findings were observed with respect to site-level factors. The coefficients of most tooth surfaces and the CAL at baseline were statistically significant for the ΔCAL value. As shown in Fig 1, for the site with a baseline CAL of 1–2 mm, the CALs were progressed, and the level of progression was highly dependent on the type of tooth, with coefficients varying from 0.06 to 0.186. All tooth type coefficients were statistically significant. Therefore, the sites with a baseline CAL of < 3 mm were generally progressed, and their changes varied among tooth types. For sites with a baseline CAL of 3 mm, the coefficients of the maxillary and mandibular molars were statistically significant and positive. Molars with a baseline CAL of 3 mm tended to be progressed. For the sites with a baseline CAL of > 3 mm, the coefficients of the maxillary molars were statistically significant and positive. Therefore, maxillary molars with a baseline CAL > 3 mm tended to be progressed. These findings were consistent with those of previous reports that have applied multilevel modeling. The coefficients for the tooth type increased from anterior to posterior teeth for the CAL progression [19] and the PPD [17], which was likely because of the inaccessibility of plaque control based on the specific anatomy of multi-rooted teeth. However, the cited studies did not classify their results by maxilla, mandible, and tooth surface. The finding that the CAL of the buccal surface of maxillary molars tended to progress is a new finding. The results from an observational study that was conducted during supportive periodontal therapy (SPT) and did not use a multilevel analysis showed that most of the lost teeth were maxillary second molars, followed by maxillary first molars, and these results reinforce our findings [11].

Few previous studies have been focused on the diagnostic value of tooth mobility, which is an interval-scale parameter; thus, individual mean values of tooth mobility are not available. Therefore, at the patient level, tooth mobility cannot be used as a dependent and/or independent variable with traditional statistical models. A mixed effects model, which can analyze hierarchical data simultaneously, can use tooth mobility as an independent variable. A previous study showed that tooth mobility is one determinant of PPD [17]. Our results showed that tooth mobility is a statistically significant factor for the ΔCAL and CAL changes. Additionally, tooth mobility was a significant predictor of burst-type progression.

Bleeding on probing (BOP) is a diagnostic criterion of gingival inflammation. Its utility as a clinical indicator for periodontal disease progression has been investigated. At the patient level, patients with a mean BOP of ≤ 20% have a significantly lower risk of CAL progression [41]. Patients with 16% or more BOP sites have a higher chance of losing attachment [42]. On the site level, periodontal pockets with successive incidence of BOP have a chance of losing attachment [42]. However, it has been shown that the positive predictive value of BOP for attachment loss is only 6%, while the negative predictive value is 98% [43]. In the present study, BOP was not a statistically significant predictor of ΔCAL. An absence of BOP may be an indicator of periodontal stability. However, further study is necessary to determine if the presence of BOP is an indicator of periodontal disease progression.

Considerable evidence has been obtained showing that periodontal pathogens are important indicators or predictors of the prognosis of periodontal disease. Several previous studies that employed mixed effects modeling have shown that among hierarchical factors, the contribution of patient-level factors was small compared with that of tooth- or site-level factors. The salivary levels of *P*. *gingivalis* and *A*. *actinomycetemcomitans* were statistically significant determinants of CAL progression, and their coefficient was not smaller than that of tooth- and site-level factors. However, for the CAL changes, the coefficient of *A*. *actinomycetemcomitans* was higher than that of the tooth- and site-level factors. Our previous report showed that the detection rate of *A*. *actinomycetemcomitans* was low. Therefore, many patients were not infected; however, carriers of *A*. *actinomycetemcomitans* were at a greater risk of CAL progression.

Two competing theories can explain the progression of periodontal disease. One theory is presented by the linear model, in which the overall sites slowly and progressively lose attachment. This theory has been supported by epidemiological studies [44, 45]. Another theory is presented by the burst model, in which multiple random sites show progression within a short period along with remission or repair. Other sites are free of progression throughout the individual’s life [32, 46–48].

Several models have been proposed to explain these dynamic conditions of periodontal disease. The “linear” model slightly overestimates changes, and the “burst” model slightly underestimates changes [49, 50]. The lack-of-fit methods could not explain the dynamic conditions [51]. Certain sites improved while others deteriorated in a cyclical manner; thus, the linear and burst theories of periodontal disease progression are a manifestation of the same phenomenon [19]. The cited studies have attempted to resolve the dynamic conditions of all sites via single equations. Therefore, these studies did not obtain conclusive results and do not provide useful information for clinicians. As described in S10 Table, both linear- and burst-type progression occurred simultaneously within the same patients, and more than half of the teeth presented burst-type progression sites accompanied by linear-type progression sites. Thus, appropriate clustering is indispensable. The results showing that the palatal surface of the maxillary premolar and the buccal surface of maxillary anterior teeth tended to exhibit burst-type progression may be useful information for dental clinicians.

This study assessed various predictors of CAL changes. In summary, the important findings were as follows:

- The baseline CAL was an important determinant of the direction of CAL changes, and the CAL changes varied widely in accordance with the tooth surface;
- Salivary levels of periodontal pathogens, such as *P*. *gingivalis* and *A*. *actinomycetemcomitans*, were affected by CAL progression;
- Linear- and burst-type progression of the CAL occurred simultaneously within the same patient, and more than half of the teeth with a burst-type progression site had an accompanying linear-type progression site; furthermore, most of the progressions were of the linear type, while the palatal surface of the maxillary premolar and buccal surface of maxillary anterior teeth tended to show burst-type progression.

## Conclusions

The parameters identified in this study may be useful to practitioners for deciding the type and extent of treatment needed at both the site and patient levels. In addition, prior hypotheses concerning "burst" and "linear" theories are not valid.

## Supporting information

S1 File Model specification. (DOCX)

S1 Table All the data analyzed in this study. (XLSX)

S2 Table Descriptive analyses of the variables analyzed in this study. (DOCX)

S3 Table The name of the ethics committees, the committee’s reference number. (DOCX)

S4 Table Descriptive analysis of the CAL change (ΔCAL) by patient, tooth and site level. (DOCX)

S5 Table Fixed effect model for ΔCAL by all the variables. (DOCX)

S6 Table Observed and predictive values of the changes in CAL between the baseline and 24 months. (DOCX)

S7 Table Models for ΔCAL by random slope of tooth type and CAL at baseline. (DOCX)

S8 Table Fixed effect model with repeated measures for the CAL changes during the 24-month follow up periods. (DOCX)

S9 Table Multilevel random effect model with repeated measures for the CAL changes during the 24-month follow up periods. (DOCX)

S10 Table Cross-tabulation of the number of “linear”- and “burst”-type progressed sites in 83 patients and 395 teeth with at least one progression site. (DOCX)

S11 Table Fixed effect model with repeated measures for the CAL changes during the 24-month follow up periods. (DOCX)

## Abbreviations

ΔCAL: Difference in CAL between the baseline and after 24 months
*A. actinomycetemcomitans*: *Aggregatibacter actinomycetemcomitans*
BOP: Bleeding on probing
CAL: Clinical attachment level
*P. gingivalis*: *Porphyromonas gingivalis*
PlI: Plaque index
PPD: Probing pocket depth
