# Supplementary material for: Site-level progression of periodontal disease during a follow-up period
Source: PLoS One. 2017 Dec 4;12(12):e0188670. doi: 10.1371/journal.pone.0188670 (PMC5714355; doi:10.1371/journal.pone.0188670)
Supplement: S1 File — (DOCX) [file pone.0188670.s001.docx]

S1 File Model Specification

**Model 1**

Multilevel random intercept model for the changes in CAL between the baseline and 24 months


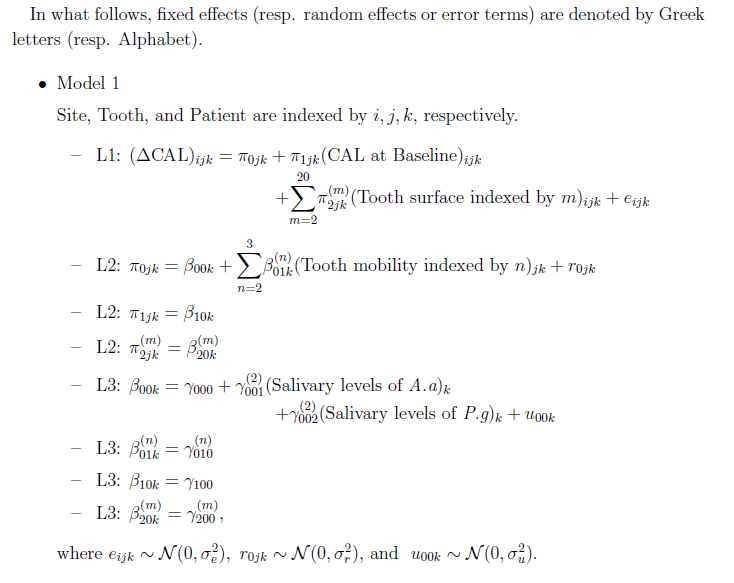


Data Structure: Patient, Tooth, Site

Random effect

Patient level: Intercept; Random effect covariance: variance component

Tooth level: Intercept; Random effect covariance: variance component

**SPSS Syntax**

MIXED ΔCAL BY Toothsurface AaBaseline PgBaseline ToothMobilityBaseline WITH CALBaseline

/CRITERIA=CIN(95) MXITER(100) MXSTEP(10) SCORING(1) SINGULAR(0.000000000001) HCONVERGE(0,ABSOLUTE) LCONVERGE(0,ABSOLUTE) PCONVERGE(0.000001, ABSOLUTE)

/FIXED= Toothsurface AaBaseline PgBaseline ToothMobilityBaseline CALBaseline | SSTYPE(3)

/METHOD=REML

/PRINT=G SOLUTION TESTCOV

/RANDOM=INTERCEPT | SUBJECT(PatientID) COVTYPE(VC)

/RANDOM=INTERCEPT | SUBJECT(PatientID*ToothID) COVTYPE(VC)

**Model 2**

Multilevel random intercept model with repeated measures for the CAL transition during the 24-month follow up periods

Model 2 (A): CAL at baseline <3mm, (B): CAL at baseline=3mm, (C) CAL at baseline >3mm

**Model 2(A) and (C)**


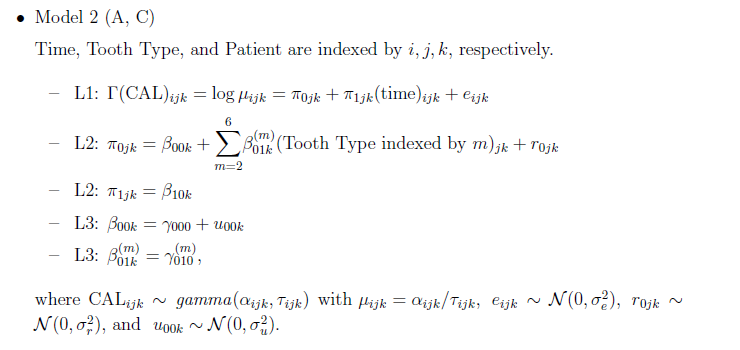


**Model 2(B)**


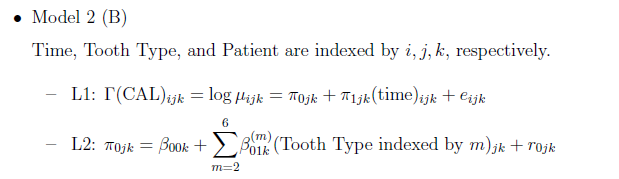


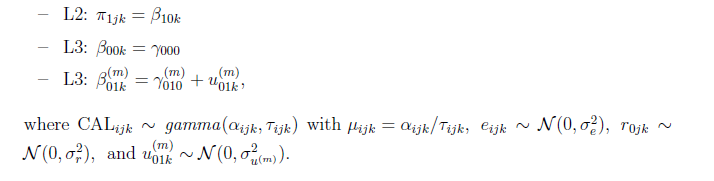


Data Structure: Patient, Tooth, Site

Repeated Measures: Time

Class of correlation structure : First Order auto regressive (AR1)

Probability distribution : Gamma

Link function: Logit

Random effect

Tooth level

For CAL at baseline <3mm or >3mm (Model 2(A) and Model 2 (C))

Intercept

Random effect covariance: variance component

For CAL at baseline =3mm (Model 2(B))

Tooth surface (Random slope)

Random effect covariance: variance component

**SPSS Syntax**

**Model 2 (A) and Model 2 (C)**

GENLINMIXED

/DATA_STRUCTURE SUBJECTS=PatientID*ToothID*SiteID REPEATED_MEASURES=Time COVARIANCE_TYPE=AR1

/FIELDS TARGET=CAL TRIALS=NONE OFFSET=NONE

/TARGET_OPTIONS DISTRIBUTION=GAMMA LINK=LOG

/FIXED EFFECTS=Time ToothType USE_INTERCEPT=TRUE

/RANDOM USE_INTERCEPT=TRUE SUBJECTS=ToothID COVARIANCE_TYPE=VARIANCE_COMPONENTS

/BUILD_OPTIONS TARGET_CATEGORY_ORDER=ASCENDING INPUTS_CATEGORY_ORDER=ASCENDING MAX_ITERATIONS=100

CONFIDENCE_LEVEL=95 DF_METHOD=RESIDUAL COVB=MODEL PCONVERGE=0.000001(ABSOLUTE) SCORING=0

SINGULAR=0.000000000001

/EMMEANS_OPTIONS SCALE=ORIGINAL PADJUST=LSD

**Model 2 (B)**

GENLINMIXED

/DATA_STRUCTURE SUBJECTS=PatientID*ToothID*SiteID REPEATED_MEASURES=Time COVARIANCE_TYPE=AR1

/FIELDS TARGET=CAL TRIALS=NONE OFFSET=NONE

/TARGET_OPTIONS DISTRIBUTION=GAMMA LINK=LOG

/FIXED EFFECTS=Time ToothType USE_INTERCEPT=TRUE

/RANDOM EFFECTS=ToothType USE_INTERCEPT=FALSE SUBJECTS=ToothID

COVARIANCE_TYPE=VARIANCE_COMPONENTS

/BUILD_OPTIONS TARGET_CATEGORY_ORDER=ASCENDING INPUTS_CATEGORY_ORDER=ASCENDING MAX_ITERATIONS=100

CONFIDENCE_LEVEL=95 DF_METHOD=RESIDUAL COVB=MODEL PCONVERGE=0.000001(ABSOLUTE) SCORING=0

SINGULAR=0.000000000001

/EMMEANS_OPTIONS SCALE=ORIGINAL PADJUST=LSD

**Model 3**

Multilevel logistic regression model with repeated measures to distinguish “linear” and “burst” progression during the 24-month follow-up periods


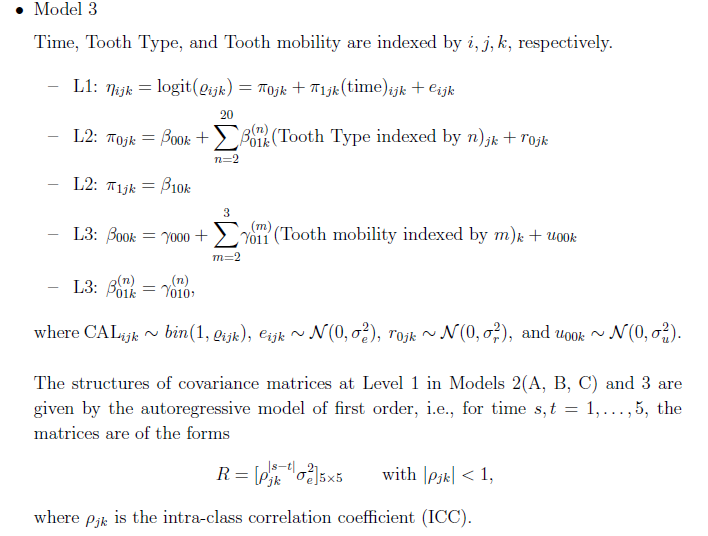


Data Structure: Patient, Tooth, Site

Repeated Measures: Time

Repeated Measures Type: First Order auto regressive (AR1)

Probability distribution: Binomial

Link function: Logit

Random effect

Patients

Intercept

Random effect covariance: variance component

Tooth

Intercept

Random effect covariance: variance component

**SPSS Syntax**

/DATA_STRUCTURE SUBJECTS=PatientID*ToothID*PocletID REPEATED_MEASURES=Time COVARIANCE_TYPE=AR1

/FIELDS TARGET=BurstLinear TRIALS=NONE OFFSET=NONE

/TARGET_OPTIONS DISTRIBUTION=BINOMIAL LINK=LOGIT

/FIXED EFFECTS=Time Tooth Movility USE_INTERCEPT=TRUE

/RANDOM USE_INTERCEPT=TRUE SUBJECTS=PatientID COVARIANCE_TYPE=VARIANCE_COMPONENTS

/RANDOM USE_INTERCEPT=TRUE SUBJECTS=IDPatient*Tooth IDCOVARIANCE_TYPE=VARIANCE_COMPONENTS

/BUILD_OPTIONS TARGET_CATEGORY_ORDER=ASCENDING INPUTS_CATEGORY_ORDER=ASCENDING MAX_ITERATIONS=100

CONFIDENCE_LEVEL=95 DF_METHOD=RESIDUAL COVB=MODEL PCONVERGE=0.000001(ABSOLUTE) SCORING=0

SINGULAR=0.000000000001

/EMMEANS_OPTIONS SCALE=ORIGINAL PADJUST=LSD.

π_0_: Site level intercept

π_1,2..._: Site level coefficient

*i*: Site level ID

ε: Site level random effect

β_0_: Tooth level intercept

β_1,2..._: Tooth level coefficient

*j*: Tooth level ID

r : Tooth level random effect

γ_0_: Subject level intercept

γ_1,2..._: Subject level coefficient

*κ*: Subject level ID

μ: Subject level random effect

CAL: Clinical attachment level

*A. a*: *Aggregatibacter actinomycetemcomitans*;

*P. g*: *Porphyromonas gingivalis*
