# Supplementary material for: Site-level progression of periodontal disease during a follow-up period
Source: PLoS One. 2017 Dec 4;12(12):e0188670. doi: 10.1371/journal.pone.0188670 (PMC5714355; doi:10.1371/journal.pone.0188670)
Supplement: S2 Table — (DOCX) [file pone.0188670.s003.docx]

**S2Table Descriptive analysis of the variables used in this study**

1. **Frequency table of CAL, Tooth mobility and BOP**

|  | | | **Baseline** | | **6 month** | | **12 month** | | **18 month** | | **24 month** | |
| --- | --- | --- | --- | --- | --- | --- | --- | --- | --- | --- | --- | --- |
|  |  |  | **n** | **%** | **n** | **%** | **n** | **%** | **n** | **%** | **n** | **%** |
| **Tooth level** | **Tooth mobility** | **0** | 16968 | 90.1 | 16920 | 89.8 | 17070 | 90.6 | 17196 | 91.3 | 17070 | 90.6 |
|  |  | **1** | 1722 | 9.1 | 1776 | 9.4 | 1602 | 8.5 | 1530 | 8.1 | 1638 | 8.7 |
|  |  | **2** | 138 | 0.7 | 126 | 0.7 | 156 | 0.8 | 102 | 0.5 | 120 | 0.6 |
|  |  | **3** | 6 | 0.0 | 12 | 0.1 | 6 | 0.0 | 6 | 0.0 | 6 | 0.0 |
| **Site level** | **CAL** | **1mm** | 1788 | 9.5 | 1717 | 9.1 | 1637 | 8.7 | 1706 | 9.1 | 1642 | 8.7 |
|  |  | **2mm** | 6550 | 34.8 | 6394 | 33.9 | 6218 | 33.0 | 6097 | 32.4 | 6230 | 33.1 |
|  |  | **3mm** | 5601 | 29.7 | 5754 | 30.6 | 5841 | 31.0 | 5946 | 31.6 | 5862 | 31.1 |
|  |  | **4mm** | 2560 | 13.6 | 2650 | 14.1 | 2745 | 14.6 | 2701 | 14.3 | 2709 | 14.4 |
|  |  | **5mm** | 1368 | 7.3 | 1387 | 7.4 | 1317 | 7.0 | 1362 | 7.2 | 1318 | 7.0 |
|  |  | **6-10mm** | 961 | 5.1 | 926 | 4.9 | 1064 | 5.6 | 1007 | 5.3 | 1059 | 5.6 |
|  |  | **10mm<** | 6 | 0.0 | 6 | 0.0 | 10 | 0.1 | 10 | 0.1 | 14 | 0.1 |
|  | **BOP** | **-** | 17281 | 91.8 | 17224 | 91.5 | 17113 | 90.9 | 17370 | 92.2 | 17231 | 91.5 |
|  |  | **+** | 1553 | 8.2 | 1610 | 8.5 | 1721 | 9.1 | 1464 | 7.8 | 1603 | 8.5 |

1. **Descriptive analysis of Individual level factors**

|  | | **Baseline** | **6 month** | **12 month** | **18 month** | **24 month** |
| --- | --- | --- | --- | --- | --- | --- |
| **PlI**  (Individual Mean) | **Mean** | 0.218 | 0.255 | 0.223 | 0.234 | 0.233 |
|  | **SD** | 0.206 | 0.231 | 0.201 | 0.199 | 0.222 |
|  | **Median** | 0.152 | 0.198 | 0.163 | 0.194 | 0.170 |
| **Salivary levels of *A. a*** | **Mean** | 0.001 | 0.000 | 0.000 | 0.000 | 0.000 |
|  | **SD** | 0.003 | 0.002 | 0.001 | 0.002 | 0.002 |
|  | **Median** | 0 | 0 | 0 | 0 | 0 |
| **Salivary levels of *P. g*** | **Mean** | 0.024 | 0.030 | 0.032 | 0.024 | 0.016 |
|  | **SD** | 0.091 | 0.080 | 0.082 | 0.054 | 0.040 |
|  | **Median** | 0.003 | 0.005 | 0.006 | 0.005 | 0 |

1. **Percentile of the Individual level factors**

|  | | **5** | **10** | **25** | **50** | **75** | **90** | **95** |
| --- | --- | --- | --- | --- | --- | --- | --- | --- |
| **PlI**  **(Individual Mean)** | **Baseline** | 0 | 0.016 | 0.070 | 0.152 | 0.324 | 0.519 | 0.682 |
|  | **6 month** | 0.012 | 0.040 | 0.093 | 0.198 | 0.372 | 0.527 | 0.625 |
|  | **12 month** | 0 | 0.020 | 0.081 | 0.163 | 0.314 | 0.506 | 0.660 |
|  | **18 month** | 0 | 0.011 | 0.066 | 0.194 | 0.363 | 0.520 | 0.614 |
|  | **24 month** | 0.018 | 0.028 | 0.074 | 0.170 | 0.321 | 0.510 | 0.658 |
| **Salivary levels of *A. a*** | **Baseline** | 0 | 0 | 0 | 0 | 0 | 0.001 | 0.004 |
|  | **6 month** | 0 | 0 | 0 | 0 | 0 | 0 | 0 |
|  | **12 month** | 0 | 0 | 0 | 0 | 0 | 0 | 0.001 |
|  | **18 month** | 0 | 0 | 0 | 0 | 0 | 0 | 0.001 |
|  | **24 month** | 0 | 0 | 0 | 0 | 0 | 0 | 0.001 |
| **Salivary levels of *P. g*** | **Baseline** | 0 | 0 | 0 | 0.003 | 0.013 | 0.054 | 0.084 |
|  | **6 month** | 0 | 0 | 0 | 0.005 | 0.025 | 0.075 | 0.106 |
|  | **12 month** | 0 | 0 | 0 | 0.006 | 0.022 | 0.100 | 0.138 |
|  | **18 month** | 0 | 0 | 0 | 0.005 | 0.021 | 0.069 | 0.107 |
|  | **24 month** | 0 | 0 | 0 | 0 | 0.011 | 0.055 | 0.086 |

1. **Distribution of ΔCAL (CAL at 24 month –CAL at baseline)**

| ∆CAL | **N** | **%** |
| --- | --- | --- |
| **<-6mm** | 7 | 0.04 |
| **-5mm** | 7 | 0.04 |
| **-4mm** | 49 | 0.26 |
| **-3mm** | 139 | 0.74 |
| **-2mm** | 718 | 3.81 |
| **-1mm** | 3128 | 16.61 |
| **0mm** | 10052 | 53.37 |
| **1mm** | 3574 | 18.98 |
| **2mm** | 800 | 4.25 |
| **3mm** | 214 | 1.14 |
| **4mm** | 88 | 0.47 |
| **5mm** | 38 | 0.20 |
| **6mm<** | 20 | 0.11 |
