# Supplementary material for: Site-level progression of periodontal disease during a follow-up period
Source: PLoS One. 2017 Dec 4;12(12):e0188670. doi: 10.1371/journal.pone.0188670 (PMC5714355; doi:10.1371/journal.pone.0188670)
Supplement: S3 Table — (DOCX) [file pone.0188670.s004.docx]

**S3　Table　The name of the ethics committees, the committee’s reference number**

| Institution | The name of the ethics committee | The committee’s reference number |
| --- | --- | --- |
| Niigata University | The regional ethical committee of the Faculty of Dentistry, Niigata University | 20-R17-08-06 |
| Keio University | Keio University School of Medicine, Ethics Committee | 20080096 |
| Hokkaido University | Institutional Review Board for Clinical Research of Hokkaido University Hospital | 008-0113 |
| Ohu University | Ohu University Research Ethics Committee | 52 |
| School of life Dentistry at Niigata, The Nippon Dental University | The Ethical Review Committee of The Nippon Dental University School of Life Dentistry at Niigata | 院151 |
| Tokyo Dental College | Ethics Committee of Tokyo Dental College | 208 |
| Bunkyo-Dori Dental Clinic | The regional ethical committee of the Faculty of Dentistry, Niigata University | 20-R17-08-06 |
| Nihon University School of Dentistry at Matsudo | Ethics Committee in Nihon University School of Dentistry at Matsudo | EC 08-014 |
| Tokyo Medical and Dental University | Dental Research Ethics Committee of Tokyo Medical and Dental University | 660 |
| Nihon University School of Dentistry | Ethical Committee of Nihon University School of Dentistry | EP08D016 |
| School of Life Dentistry at Tokyo, The Nippon Dental University | The Institutional Review Board of Nippon Dental University. | 2・１・22 |
| Matsumoto Dental University | The Ethics Committee of Matsumoto Dental University | 0090 |
| Aichi Gakuin University | Ethics　Committee　：Aichi　Gakuin University，School of Dentistry | 158 |
| Osaka Dental University | the Ethics Committee of Osaka Dental University | 80712 |
| Kyushu University | Ethical Committee of Kyushu University Faculty of Dental Science | No. 20 - 11 |
| Nagasaki University | The Ethics Committee, Nagasaki University Graduate School of Biomedical Sciences | 0846-2 |
| Kagoshima University | Ethical Committee of Kagoshima University Medical and Dental Hospital. | 20-58 |
