# Supplementary material for: Site-level progression of periodontal disease during a follow-up period
Source: PLoS One. 2017 Dec 4;12(12):e0188670. doi: 10.1371/journal.pone.0188670 (PMC5714355; doi:10.1371/journal.pone.0188670)
Supplement: S4 Table — (DOCX) [file pone.0188670.s005.docx]

**S4Table Descriptive analysis of the CAL change (**∆**CAL) by patient, tooth and site level**

**(A) Patient-level cross-tabulation of improved and progressed CAL from the baseline to after 24 months**

|  | | Number of sites: ∆CAL≥3 mm | | | | | | | |
| --- | --- | --- | --- | --- | --- | --- | --- | --- | --- |
|  |  | **0** | **1** | **2** | **3** | **4** | **5-9** | **10**≥ | **Total** |
| Number of sites: ∆CAL≤−3 mm | **0** | 40 | 7 | 5 | 3 | 4 | 9 | 2 | 70 |
|  | **1** | 5 | 1 | 2 | 3 | 2 | 2 | 2 | 17 |
|  | **2** | 4 | 0 | 1 | 2 | 1 | 1 | 1 | 10 |
|  | **3** | 2 | 1 | 1 | 0 | 0 | 3 | 1 | 8 |
|  | **4** | 3 | 0 | 1 | 0 | 0 | 1 | 2 | 7 |
|  | **5-9** | 5 | 1 | 0 | 1 | 0 | 0 | 1 | 8 |
|  | **10**≥ | 2 | 0 | 0 | 0 | 0 | 1 | 1 | 4 |
|  | **Total** | 61 | 10 | 10 | 9 | 7 | 17 | 19 | 124 |

CAL: clinical attachment level; ΔCAL: difference between the baseline and after 24 months

In total, 32.3% (40/124) of the patients had only stable sites, 16.9% (21/124) had only improved sites, and 24.2% (30/124) had only progressed sites; and 26.7% (33/124) had both improved and progressed sites.

**(B) Tooth-level cross-tabulation of the number of sites with a progressed CAL from the baseline to after 24 months**

|  | | Number of sites: ∆CAL≥3 mm | | | | | | | |
| --- | --- | --- | --- | --- | --- | --- | --- | --- | --- |
|  |  | 0 | 1 | 2 | 3 | 4 | 5 | 6 | Total |
| Number of sites: ∆CAL≤−3 mm | 0 | 2807 | 125 | 38 | 25 | 5 | 2 | 5 | 3007 |
|  | 1 | 80 | 4 | 5 | 2 | 0 | 0 | 0 | 91 |
|  | 2 | 17 | 2 | 0 | 0 | 0 | 0 | 0 | 19 |
|  | 3 | 15 | 0 | 1 | 0 | 0 | 0 | 0 | 16 |
|  | 4 | 5 | 0 | 0 | 0 | 0 | 0 | 0 | 5 |
|  | 5 | 1 | 0 | 0 | 0 | 0 | 0 | 0 | 1 |
|  | 6 | 0 | 0 | 0 | 0 | 0 | 0 | 0 | 0 |
|  | Total | 2925 | 131 | 44 | 27 | 5 | 2 | 5 | 3139 |

In total, 89.2% (2807/3139) of the teeth had only stable sites, 3.4% (118/3139) had only improved sites, and 6.4% (200/3139) had only progressed sites; and 0.4% (14/3139) had both improved and progressed sites.

Thus, few teeth had both progressed and improved sites.

**(C) Site-level descriptive statistics for the difference in CAL from the baseline to after 24 months (∆CAL)**

|  | | | ∆**CAL** | | | | | | | |
| --- | --- | --- | --- | --- | --- | --- | --- | --- | --- | --- |
|  |  |  | ≤**-3 mm** | | **2 mm-2 mm** | | **3 mm**≤ | | **Total** | |
|  |  |  | **n** | **%** | **n** | **%** | **n** | **%** | **n** | **%** |
| **Subject-level explanatory variable** | | | | | | | | | | |
| **Salivary levels of *A. a*** | | **0.00006%**≤ | 198 | 1.1% | 16731 | 97.1% | 309 | 1.8% | 17238 | 100% |
|  |  | **<0.00006%** | 4 | 0.3% | 1541 | 96.6% | 51 | 3.2% | 1596 | 100% |
| **Salivary levels of *P. g*** | | **0.0067%**≤ | 119 | 1.0% | 12197 | 97.8% | 152 | 1.2% | 12468 | 100% |
|  |  | **<0.0067%** | 83 | 1.3% | 6075 | 95.4% | 208 | 3.3% | 6366 | 100% |
| **Tooth-level explanatory variable** | | | | | | | | | | |
| **Tooth mobility** | | **0** | 181 | 1.1% | 16478 | 97.1% | 309 | 1.8% | 16968 | 100% |
|  |  | **1** | 17 | 1.0% | 1664 | 96.6% | 41 | 2.4% | 1722 | 100% |
|  |  | **2-3** | 4 | 2.8% | 130 | 90.3% | 10 | 6.9% | 144 | 100% |
| **PlI**  (Tooth Mean) | | ≤**0.1%** | 3324 | 46.7% | 1913 | 26.9% | 1885 | 26.5% | 7122 | 100% |
|  |  | **0.1-0.2%** | 1634 | 44.4% | 1017 | 27.7% | 1027 | 27.9% | 3678 | 100% |
|  |  | **0.2%**≤ | 3380 | 42.1% | 2671 | 33.2% | 1983 | 24.7% | 8034 | 100% |
| **Site-level explanatory variable** | | | | | | | | | | |
| **CAL at baseline** | | ≤**2 mm** | 0 | 0% | 8139 | 97.6% | 199 | 2.4% | 8338 | 100% |
|  |  | **3 mm** | 0 | 0% | 5527 | 98.7% | 74 | 1.3% | 5601 | 100% |
|  |  | **4 mm**≤ | 202 | 4.1% | 4606 | 94.1% | 87 | 1.8% | 4895 | 100% |
| **BOP** | | **-** | 160 | 0.9% | 16806 | 97.3% | 315 | 1.8% | 17281 | 100% |
|  |  | **+** | 42 | 2.7% | 1466 | 94.4% | 45 | 2.9% | 1553 | 100% |
| **Tooth surface** | | | | | | | | | | |
| **Mandibular** | **Anterior** | **Lingual** | 12 | 1.6% | 714 | 97.0% | 10 | 1.4% | 736 | 100% |
|  |  | **Labial** | 9 | 1.2% | 713 | 97.7% | 8 | 1.1% | 730 | 100% |
|  |  | **Approximal** | 39 | 1.3% | 2818 | 96.8% | 55 | 1.9% | 2912 | 100% |
|  | **Premolar** | **Lingual** | 1 | 0.2% | 452 | 98.7% | 5 | 1.1% | 458 | 100% |
|  |  | **Buccal** | 8 | 1.7% | 439 | 95.9% | 11 | 2.4% | 458 | 100% |
|  |  | **Approximal** | 11 | 0.6% | 1772 | 98.2% | 21 | 1.2% | 1804 | 100% |
|  | **Molar** | **Lingual** | 8 | 2.0% | 385 | 94.6% | 14 | 3.4% | 407 | 100% |
|  |  | **Buccal** | 9 | 2.2% | 384 | 94.3% | 14 | 3.4% | 407 | 100% |
|  |  | **Approximal** | 21 | 1.8% | 1110 | 95.5% | 31 | 2.7% | 1162 | 100% |
|  |  | **Distal** | 11 | 2.2% | 472 | 95.2% | 13 | 2.6% | 496 | 100% |
| **Maxillary** | **Anterior** | **Lingual** | 3 | 0.4% | 699 | 99.3% | 2 | 0.3% | 704 | 100% |
|  |  | **Labial** | 7 | 1.0% | 691 | 98.2% | 6 | 0.9% | 704 | 100% |
|  |  | **Approximal** | 14 | 0.5% | 2775 | 98.8% | 19 | 0.7% | 2808 | 100% |
|  | **Premolar** | **Palatal** | 3 | 0.7% | 447 | 98.2% | 5 | 1.1% | 455 | 100% |
|  |  | **Buccal** | 4 | 0.9% | 445 | 97.8% | 6 | 1.3% | 455 | 100% |
|  |  | **Approximal** | 17 | 1.0% | 1734 | 97.3% | 31 | 1.7% | 1782 | 100% |
|  | **Molar** | **Palatal** | 4 | 1.0% | 369 | 95.8% | 12 | 3.1% | 385 | 100% |
|  |  | **Buccal** | 1 | 0.3% | 365 | 94.8% | 19 | 4.9% | 385 | 100% |
|  |  | **Approximal** | 11 | 1.0% | 1026 | 94.1% | 53 | 4.9% | 1090 | 100% |
|  |  | **Distal** | 9 | 1.8% | 462 | 93.1% | 25 | 5.0% | 496 | 100% |
