# Supplementary material for: Site-level progression of periodontal disease during a follow-up period
Source: PLoS One. 2017 Dec 4;12(12):e0188670. doi: 10.1371/journal.pone.0188670 (PMC5714355; doi:10.1371/journal.pone.0188670)
Supplement: S5 Table — (DOCX) [file pone.0188670.s006.docx]

**S5 Table Fixed Effect model for ∆CAL by all the variables**

PlI and BOP were not statistically significant.

**Model specification**

Grouping variable: Patient, Tooth

**SPSS Syntax**

MIXED DeltaCAL BY ToothTypeSurface BOPR AaR PgR MovilityR WITH CALBaseline PlItiithmean

/CRITERIA=CIN(95) MXITER(200) MXSTEP(10) SCORING(1) SINGULAR(0.000000000001) HCONVERGE(0,ABSOLUTE) LCONVERGE(0, ABSOLUTE) PCONVERGE(0.000001, ABSOLUTE)

/FIXED=ToothTypeSurface BOPR AaR PgR MovilityR CALBaseline PlItiithmean | SSTYPE(3)

/METHOD=REML

/PRINT=G SOLUTION TESTCOV
