# Supplementary material for: Site-level progression of periodontal disease during a follow-up period
Source: PLoS One. 2017 Dec 4;12(12):e0188670. doi: 10.1371/journal.pone.0188670 (PMC5714355; doi:10.1371/journal.pone.0188670)
Supplement: S6 Table — (DOCX) [file pone.0188670.s007.docx]

**S6Table Observed and predictive values of the changes in CAL between the baseline and 24 months**

|  | | | | Observed value (∆CAL) | | |  | Predictive value | | |
| --- | --- | --- | --- | --- | --- | --- | --- | --- | --- | --- |
|  |  |  |  | mean | SD | SE |  | mean | SD | SE |
|  | **Subject-level explanatory variable** | | | | | | | | | |
| Salivary levels of *A. a* | | | 0.00006%≥ | 0.043 | 1.040 | 0.008 |  | 0.043 | 0.640 | 0.005 |
|  |  |  | ≥0.00006% | 0.306 | 1.046 | 0.026 |  | 0.306 | 0.649 | 0.016 |
| Salivary levels of *P. g* | | | 0.0067%≥ | 0.021 | 0.969 | 0.009 |  | 0.021 | 0.597 | 0.005 |
|  |  |  | ≥0.0067% | 0.153 | 1.170 | 0.015 |  | 0.153 | 0.723 | 0.009 |
|  | **Tooth-level explanatory variable** | | | | | | | | | |
| Tooth mobility | | | 0 | 0.061 | 1.029 | 0.008 |  | 0.061 | 0.638 | 0.005 |
|  |  |  | 1 | 0.086 | 1.114 | 0.027 |  | 0.086 | 0.691 | 0.017 |
|  |  |  | 2－3 | 0.264 | 1.664 | 0.139 |  | 0.264 | 0.882 | 0.074 |
|  | **Site-level explanatory variable** | | | | | | | | | |
| CAL at Baseline | | | 2 mm≥ | 0.383 | 0.842 | 0.009 |  | 0.359 | 0.486 | 0.005 |
|  |  |  | 3 mm | 0.018 | 0.890 | 0.012 |  | 0.048 | 0.512 | 0.007 |
|  |  |  | ≥ 4 mm | -0.422 | 1.289 | 0.018 |  | -0.416 | 0.721 | 0.010 |
| Tooth surface | | | | | | | | | | |
| Mandibular | | Anterior | Lingual | -0.001 | 1.057 | 0.039 |  | -0.001 | 0.665 | 0.025 |
|  |  |  | Labial | 0.115 | 0.989 | 0.037 |  | 0.115 | 0.657 | 0.024 |
|  |  |  | Approximal | 0.014 | 1.046 | 0.019 |  | 0.014 | 0.681 | 0.013 |
|  |  | Premolar | Lingual | 0.063 | 0.923 | 0.043 |  | 0.063 | 0.571 | 0.027 |
|  |  |  | Buccal | 0.124 | 1.121 | 0.052 |  | 0.124 | 0.633 | 0.030 |
|  |  |  | Approximal | 0.042 | 0.944 | 0.022 |  | 0.042 | 0.579 | 0.014 |
|  |  | Molar | Lingual | 0.115 | 1.166 | 0.058 |  | 0.115 | 0.725 | 0.036 |
|  |  |  | Buccal | 0.047 | 1.238 | 0.061 |  | 0.047 | 0.708 | 0.035 |
|  |  |  | Approximal | 0.055 | 1.131 | 0.033 |  | 0.055 | 0.671 | 0.020 |
|  |  |  | Distal | 0.107 | 1.136 | 0.051 |  | 0.107 | 0.701 | 0.031 |
| Maxillary | | Anterior | Lingual | 0.018 | 0.712 | 0.027 |  | 0.018 | 0.454 | 0.017 |
|  |  |  | Labial | 0.038 | 0.944 | 0.036 |  | 0.038 | 0.558 | 0.021 |
|  |  |  | Approximal | 0.044 | 0.865 | 0.016 |  | 0.044 | 0.532 | 0.010 |
|  |  | Premolar | Palatal | 0.051 | 0.931 | 0.044 |  | 0.051 | 0.474 | 0.022 |
|  |  |  | Buccal | 0.090 | 1.025 | 0.048 |  | 0.090 | 0.623 | 0.029 |
|  |  |  | Approximal | 0.072 | 1.015 | 0.024 |  | 0.072 | 0.605 | 0.014 |
|  |  | Molar | Palatal | 0.190 | 1.251 | 0.064 |  | 0.190 | 0.773 | 0.039 |
|  |  |  | Buccal | 0.332 | 1.320 | 0.067 |  | 0.332 | 0.797 | 0.041 |
|  |  |  | Approximal | 0.128 | 1.329 | 0.040 |  | 0.128 | 0.809 | 0.025 |
|  |  |  | Distal | 0.127 | 1.323 | 0.059 |  | 0.127 | 0.833 | 0.037 |
