# Supplementary material for: Site-level progression of periodontal disease during a follow-up period
Source: PLoS One. 2017 Dec 4;12(12):e0188670. doi: 10.1371/journal.pone.0188670 (PMC5714355; doi:10.1371/journal.pone.0188670)
Supplement: S7 Table — (DOCX) [file pone.0188670.s008.docx]

**S7 Table Models for ∆CAL by random slope of tooth type and CAL at baseline**

1. **Random effect model by tooth type**

1. **Random effect model by CAL at baseline**

Model specification

Grouping variable: Patient, Tooth

Fixed effect: Tooth type (Mandibular anterior, Mandibular premolar, Mandibular molar

Maxilla anterior, Maxilla premolar, Maxilla molar), CAL at baseline (<3mm, 3mm, >3mm)

Random effect:

Patient level: Intercept, Random effect covariance: variance component

Tooth level: tooth type or CAL (Random slope), Random effect covariance: unstructured

**SPSS Syntax**

**(A)**

MIXED DeltaCAL BY CALClassification ToothType

/CRITERIA=CIN(95) MXITER(200) MXSTEP(10) SCORING(1) SINGULAR(0.000000000001) HCONVERGE(0, ABSOLUTE) LCONVERGE(0, ABSOLUTE) PCONVERGE(0.000001, ABSOLUTE)

/FIXED=CALClassification ToothType | SSTYPE(3)

/METHOD=REML

/PRINT=G SOLUTION TESTCOV

/RANDOM=INTERCEPT | SUBJECT(PatientID) COVTYPE(VC)

/RANDOM= ToothType | SUBJECT(PatientID*toothID) COVTYPE(UN)

**(B)**

MIXED DeltaCAL BY CALBaselineType ToothType

/CRITERIA=CIN(95) MXITER(200) MXSTEP(10) SCORING(1) SINGULAR(0.000000000001) HCONVERGE(0, ABSOLUTE) LCONVERGE(0, ABSOLUTE) PCONVERGE(0.000001, ABSOLUTE)

/FIXED=CALBaselineType ToothType | SSTYPE(3)

/METHOD=REML

/PRINT=G SOLUTION TESTCOV

/RANDOM=INTERCEPT | SUBJECT(PatientID) COVTYPE(VC)

/RANDOM=CALClassification | SUBJECT(PatientID*toothID) COVTYPE(UN)
