# Supplementary material for: Site-level progression of periodontal disease during a follow-up period
Source: PLoS One. 2017 Dec 4;12(12):e0188670. doi: 10.1371/journal.pone.0188670 (PMC5714355; doi:10.1371/journal.pone.0188670)
Supplement: S8 Table — (DOCX) [file pone.0188670.s009.docx]

**S8 Table Fixed effect model with repeated measures for the CAL changes during the 24-month follow up periods**

**Model specification**

Data Structure: Patient, Tooth, Site

Repeated Measures: Time

Class of correlation structure: First Order auto regressive (AR1)

Probability distribution: Gamma

Link function: Logit

**SPSS Syntax**

GENLINMIXED

/DATA_STRUCTURE SUBJECTS=PatientID*ToothID*SiteID REPEATED_MEASURES=Time COVARIANCE_TYPE=AR1

/FIELDS TARGET=CAL TRIALS=NONE OFFSET=NONE

/TARGET_OPTIONS DISTRIBUTION=GAMMA LINK=LOG

/FIXED EFFECTS=Time Aa Pg PlI BOPR ToothMovilityR ToothTypeSurface USE_INTERCEPT=TRUE

/BUILD_OPTIONS TARGET_CATEGORY_ORDER=ASCENDING INPUTS_CATEGORY_ORDER=ASCENDING MAX_ITERATIONS=100

CONFIDENCE_LEVEL=95 DF_METHOD=RESIDUAL COVB=MODEL PCONVERGE=0.000001(ABSOLUTE) SCORING=0 SINGULAR=0.000000000001 /EMMEANS_OPTIONS SCALE=ORIGINAL PADJUST=LSD.
