# Supplementary material for: Site-level progression of periodontal disease during a follow-up period
Source: PLoS One. 2017 Dec 4;12(12):e0188670. doi: 10.1371/journal.pone.0188670 (PMC5714355; doi:10.1371/journal.pone.0188670)
Supplement: S9 Table — (DOCX) [file pone.0188670.s010.docx]

**S9 Table Multilevel random effect model with repeated measures for the CAL changes during the 24-month follow up periods**

**Model by the variable of combination tooth type and tooth surface**

**Model specification**

Data Structure: Patient, Tooth, Site

Repeated Measures: Time

Class of correlation structure: First Order auto regressive (AR1)

Probability distribution: Gamma

Link function: Logit

Random effect

Tooth level

For CAL at baseline <3mm or >3mm (Model S8 (A) and Model S8 (C))

Intercept

Random effect covariance: variance component

For CAL at baseline =3mm (Model S8 (B))

Tooth surface (Random slope)

Random effect covariance: variance component

**SPSS Syntax**

**Model 8(A) and (C)**

GENLINMIXED

/DATA_STRUCTURE SUBJECTS=PatientID*ToothID*SiteID REPEATED_MEASURES=Time COVARIANCE_TYPE=AR1

/FIELDS TARGET=CAL TRIALS=NONE OFFSET=NONE

/TARGET_OPTIONS DISTRIBUTION=GAMMA LINK=LOG

/FIXED EFFECTS=Time ToothTypeSurface USE_INTERCEPT=TRUE

/RANDOM USE_INTERCEPT=TRUE SUBJECTS=ToothID COVARIANCE_TYPE=VARIANCE_COMPONENTS

/BUILD_OPTIONS TARGET_CATEGORY_ORDER=ASCENDING INPUTS_CATEGORY_ORDER=ASCENDING MAX_ITERATIONS=100

CONFIDENCE_LEVEL=95 DF_METHOD=RESIDUAL COVB=MODEL PCONVERGE=0.000001(ABSOLUTE) SCORING=0

SINGULAR=0.000000000001

/EMMEANS_OPTIONS SCALE=ORIGINAL PADJUST=LSD.

**Model 8(B)**

GENLINMIXED

/DATA_STRUCTURE SUBJECTS=PatientID*ToothID*SiteID REPEATED_MEASURES=Time COVARIANCE_TYPE=AR1

/FIELDS TARGET=CAL TRIALS=NONE OFFSET=NONE

/TARGET_OPTIONS DISTRIBUTION=GAMMA LINK=LOG

/FIXED EFFECTS=Time ToothTypeSurface USE_INTERCEPT=TRUE

/RANDOM EFFECTS=ToothTypeSurface USE_INTERCEPT=FALSE SUBJECTS=ToothID

COVARIANCE_TYPE=VARIANCE_COMPONENTS

/BUILD_OPTIONS TARGET_CATEGORY_ORDER=ASCENDING INPUTS_CATEGORY_ORDER=ASCENDING MAX_ITERATIONS=100

CONFIDENCE_LEVEL=95 DF_METHOD=RESIDUAL COVB=MODEL PCONVERGE=0.000001(ABSOLUTE) SCORING=0

SINGULAR=0.000000000001
