# Supplementary material for: Site-level progression of periodontal disease during a follow-up period
Source: PLoS One. 2017 Dec 4;12(12):e0188670. doi: 10.1371/journal.pone.0188670 (PMC5714355; doi:10.1371/journal.pone.0188670)
Supplement: S10 Table — (DOCX) [file pone.0188670.s011.docx]

**S10 Table Cross-tabulation of the number of “linear”- and “burst”-type progressed sites in 83 patients and 395 teeth with at least one progression site**

1. **Patient-level cross tabulation**

|  | | **Number of “linear”-type progressed sites** | | | | | | | |
| --- | --- | --- | --- | --- | --- | --- | --- | --- | --- |
|  |  | **0** | **1** | **2** | **3** | **4** | **5**≤**and ＜10** | **10**≤ | **Total** |
| **Number of “burst”-type**  **Progressed sites** | **0** | 0 | 10 | 7 | 5 | 6 | 12 | 3 | 43 |
|  | **1** | 1 | 3 | 2 | 0 | 1 | 5 | 6 | 19 |
|  | **2**≤ | 0 | 0 | 0 | 1 | 0 | 8 | 10 | 21 |
|  | **Total** | 1 | 13 | 9 | 6 | 7 | 25 | 19 | 83 |

Among 83 patients with at least one progressed site, 43 (51.8%) had only “linear”-type progressed sites and 40 (51.8%) had both “linear”- and “burst”-type progressed sites. One patient had a “burst”-type progressed site but no “linear” types.

1. **Tooth-level cross-tabulation**

|  | | Number of “linear”-type progressed sites | | | | | | | |
| --- | --- | --- | --- | --- | --- | --- | --- | --- | --- |
|  |  | 0 | 1 | 2 | 3 | 4 | 5 | 6 | Total |
| Number of “burst”-type  Progressed site | 0 | 0 | 203 | 76 | 36 | 7 | 1 | 1 | 324 |
|  | 1 | 22 | 9 | 13 | 3 | 2 | 0 | 0 | 49 |
|  | 2 | 4 | 3 | 1 | 0 | 0 | 0 | 0 | 8 |
|  | 3 | 5 | 1 | 3 | 2 | 0 | 0 | 0 | 11 |
|  | 4 | 0 | 0 | 1 | 0 | 0 | 0 | 0 | 1 |
|  | 5 | 0 | 1 | 0 | 0 | 0 | 0 | 0 | 1 |
|  | 6 | 1 | 0 | 0 | 0 | 0 | 0 | 0 | 1 |
|  | Total | 32 | 217 | 94 | 41 | 9 | 1 | 1 | 395 |

Among the 71 teeth with “burst”-type progressed sites, 32 (45.1%) were without “linear” aggravation sites and 39 (54.9%) had accompanying “linear”-type progressed sites. However, because we investigated 3,139 teeth in total, those teeth were very few.
