# Supplementary material for: Site-level progression of periodontal disease during a follow-up period
Source: PLoS One. 2017 Dec 4;12(12):e0188670. doi: 10.1371/journal.pone.0188670 (PMC5714355; doi:10.1371/journal.pone.0188670)
Supplement: S11 Table — (DOCX) [file pone.0188670.s012.docx]

**S11 Table Multilevel logistic regression model with repeated measures to distinguish “linear” and “burst” progression during the 24-month follow-up periods by all of the variable used in this study**

**(Fixed effect model)**

**Model specification**

Data Structure: Patient, Tooth, Site

Repeated Measures: Time

Repeated Measures Type: First Order auto regressive (AR1)

Probability distribution: Binomial

Link function: Logit

**SPSS Syntax**

GENLINMIXED

/DATA_STRUCTURE SUBJECTS=IDPatient*ToothID*PocketID REPEATED_MEASURES=Time COVARIANCE_TYPE=AR1

/FIELDS TARGET=BurstLinear TRIALS=NONE OFFSET=NONE

/TARGET_OPTIONS DISTRIBUTION=BINOMIAL LINK=LOGIT

/FIXED EFFECTS=Time Tooth Aa Pg PlI Movility BOP CALBase USE_INTERCEPT=TRUE

/BUILD_OPTIONS　TARGET_CATEGORY_ORDER=ASCENDING INPUTS_CATEGORY_ORDER=ASCENDING MAX_ITERATIONS=100

CONFIDENCE_LEVEL=95 DF_METHOD=RESIDUAL COVB=MODEL PCONVERGE=0.000001(ABSOLUTE) SCORING=0

SINGULAR=0.000000000001

/EMMEANS_OPTIONS SCALE=ORIGINAL PADJUST=LSD.
